# Scapular Muscle Activation at Different Shoulder Abduction Angles During Pilates Reformer Arm Work Exercise

**DOI:** 10.3390/medicina61040645

**Published:** 2025-04-01

**Authors:** Woo-Lim Mun, Eui-Young Jung, Shi Lei, Su-Yeon Roh

**Affiliations:** 1 Department of Exercise Rehabilitation, Gachon University, Incheon 21936, Republic of Korea; woolim@gachon.ac.kr; 2 Department of Health Science, Gachon University Graduate School, Incheon 21936, Republic of Korea

**Keywords:** electromyography, scapular muscle, shoulder abduction, Pilates reformer arm work

## Abstract

*Background and Objectives*: Scapular muscles are crucial in controlling scapular movement, ensuring proper alignment, and preventing injuries during shoulder joint motion. The shoulder abduction angle is one of the most important factors when performing exercises to improve scapular movement control. The Pilates reformer arm work (AW) movement can be performed with different shoulder abduction angles. Therefore, this study analyzed the differences in scapular muscle activation depending on the shoulder abduction angle during the AW movement. *Materials and Methods*: Twenty-six healthy adult males performed the AW movement under four shoulder abduction angle conditions (0°, 90°, 135°, 160°). When performing the AW in the four conditions, surface electromyography analyzed the muscle activation of the upper trapezius (UT), middle trapezius (MT), lower trapezius (LT), serratus anterior (SA), posterior deltoid (PD), and levator scapulae (LS), and the muscle activation ratios of the UT/LT, LS/LT, UT/SA, and LS/SA. *Results*: The UT, LT, and SA muscle activation increased proportionally with the increasing shoulder abduction angle (*p* < 0.001). The LT showed the highest muscle activity at the shoulder abduction angle of 135° (*p* < 0.001). The MT and PD showed the highest muscle activities at the shoulder abduction angle of 90° (*p* < 0.001). The muscle activity ratios of the UT/LT and LS/LT were the lowest at the shoulder abduction angles of 90° and 135° (*p* < 0.001). The muscle activity ratio between the UT/SA and LS/SA was the lowest at the 135° and 160° shoulder abduction angles (*p* < 0.001). *Conclusions*: Our findings highlight the impact of the shoulder abduction angle on scapular muscle activation, suggesting that different angles may be used to engage specific muscles during Pilates reformer arm work exercises selectively. This insight can guide exercise interventions for individuals aiming to improve their scapular control and stability.

## 1. Introduction

Pilates is a full-body workout that improves body alignment, strengthens the core, and improves flexibility and balance [1]. In particular, Pilates has been used as an effective exercise method to promote musculoskeletal health, correct posture, and improve functional movement patterns [2]. The Pilates reformer utilizes spring resistance to adjust the intensity of the exercise and helps improve whole-body muscle coordination and stability using various support surfaces and movable carriages [3]. Reformer Pilates can be more effective than mat Pilates in certain areas, such as core strength and endurance [4]. Specifically, on the Pilates reformer, arm work (AW) movements are performed to activate the surrounding muscles and strengthen the scapular control to ensure shoulder stability [5]. In addition, the AW movement can be customized to the subject’s needs by varying the shoulder abduction angle. The reason for varying the shoulder abduction angle is to effectively induce the activation of specific scapular muscles [6]. Muscle activation according to the shoulder abduction angle plays an important role in rehabilitation exercises because it directly affects joint stability [7]. Individual differences among subjects, such as variations in the shoulder joint range of motion, strength imbalances, and previous injury history, necessitate adjustments to the shoulder abduction angle [8]. Understanding these variations is essential for designing effective rehabilitation protocols that cater to individual needs and optimize recovery.

Applying a proper shoulder abduction angle is important for optimal shoulder stability and scapular control. During shoulder abduction, the trapezius (upper, middle, and lower parts), deltoid, and serratus anterior have coordinated muscle activation to produce appropriate scapular movement to maintain shoulder joint alignment [9]. However, overactivation of the muscles that elevate the scapula, such as the upper trapezius and levator scapulae, and low activation of the muscles that stabilize the scapula, such as the middle and lower trapezii and serratus anterior, can lead to increased shoulder instability, which can increase the risk of musculoskeletal conditions such as impingement syndrome and rotator cuff injury [10,11,12]. Therefore, for proper arm movement, while maintaining scapular stability, it is necessary to exercise at the shoulder abduction angle, which can prevent muscle overactivity that causes scapular elevation and induce the activation of the middle and lower trapezii and serratus anterior.

Many researchers have attempted to analyze differences in scapular muscle activation depending on the shoulder abduction angle. Previous studies have shown that as the shoulder abduction angle increased (from 0° to 150°), muscle activity in the UT and LS also increased [13,14]. The LT and SA showed the highest muscle activity in movements with shoulder abduction angles of 120° and 140°, respectively [14,15]. The muscle activity of the MT and PD was the highest at the 90° shoulder abduction angle [16,17,18]. Different shoulder abduction angles change the position of the scapula, which changes the level of function of the scapular muscles [19]. However, results have been reported with the highest arm elevation at a shoulder abduction of 150°, but the level of muscle activity associated with scapular movement at higher angles remains unclear. Therefore, it is necessary to study the level of activation of the scapular muscles when performing exercises at shoulder abduction angles greater than 150°. In clinical practice, normal shoulder abduction is considered to be 160° [20], and exercises are attempted for scapular muscle activation at a shoulder abduction angle of 160°.

This study aimed to determine the muscle activation of the UT, MT, LT, SA, PD, and LS, which are muscles that can influence scapular movement, when performing a Pilates reformer AW movement with varying shoulder abduction angles of 0°, 90°, 135°, and 160°. Furthermore, the purpose of this study was to identify and provide knowledge on the UT/LT, UT/SA, LS/LT, and LS/SA muscle activity ratios that can promote optimal scapular muscle activation.

## 2. Materials and Methods

### 2.1. Participants

This study was approved by the Gachon University Ethics Committee, and all experiments were conducted in accordance with the Declaration of Helsinki (IRB approval number: 1044396-202409-HR-152-01). We also registered with the Clinical Research Information Service (CRIS) and received a registration number (KCT number: KCT0010032). A total of 26 healthy male adults (age: 22.9 ± 1.5 years; height: 173.7 ± 5.2 cm; weight: 77.3 ± 8.6 kg; body mass index: 25.6 ± 2.4 kg/m^2^; muscle skeletal mass: 36.1 ± 3.4 kg; fat percent: 16.9 ± 5.2%; dominant limb: 22 right-sided and 4 left-sided) were recruited. All subjects confirmed their willingness to voluntarily participate in this study at the initial visit and informed consent was obtained. Inclusion criteria included (1) at least 12 months of Pilates participation experience, (2) knowing the arm work movement on the reformer, and the ability to perform without pain. Exclusion criteria included (1) shoulder pain, (2) surgery on the musculoskeletal system of the trunk and upper extremities, (3) abnormal blood pressure, (4) pain or discomfort present during movement, and (5) neurologic disease. We used G*Power software (Version 3.1.9.4, Franz Faul, Universitat Keil, Germany) to calculate the sample size required for this study. As far as we know, no other study has used a similar design, so we needed a sample size of at least 24 participants to conduct the four conditions (arm work shoulder abduction angles of 0°, 90°, 135°, and 160°), with a power of 0.80, an alpha error probability of 0.05, and an effect size of 0.25 (Cohen’s f medium effect size) [21]. However, we recruited 27 participants to account for a 10% dropout. One participant complained of discomfort and stopped the measurement. So, 26 participants were finally included in the analysis.

### 2.2. Experimental Procedures

This study was a single-group, four-condition, repeated-measures design. This investigation contributes to the knowledge of electromyography (EMG) at the concentric (CON), isometric (ISO), and eccentric (ECC) phases during Pilates arm work (AW) movements, based on differences in the shoulder abduction angles of 0° (AW0), 90° (AW90), 135° (AW135), and 160° (AW160). All participants were asked to refrain from strenuous physical activity and stimulant-containing supplements on the day of the study and the day before. Each participant attended three sessions separated by at least 48 h. All scheduling was performed at the Integrative Movement Science Laboratory (IMSL). During the first and second sessions, all participants were familiarized with the AW movements on a Pilates apparatus, reformer, and variable-resistance spring (Balanced Body Inc., Sacramento, CA, USA). In the third session, we collected demographic and EMG data for each participant.

**Figure 1:**
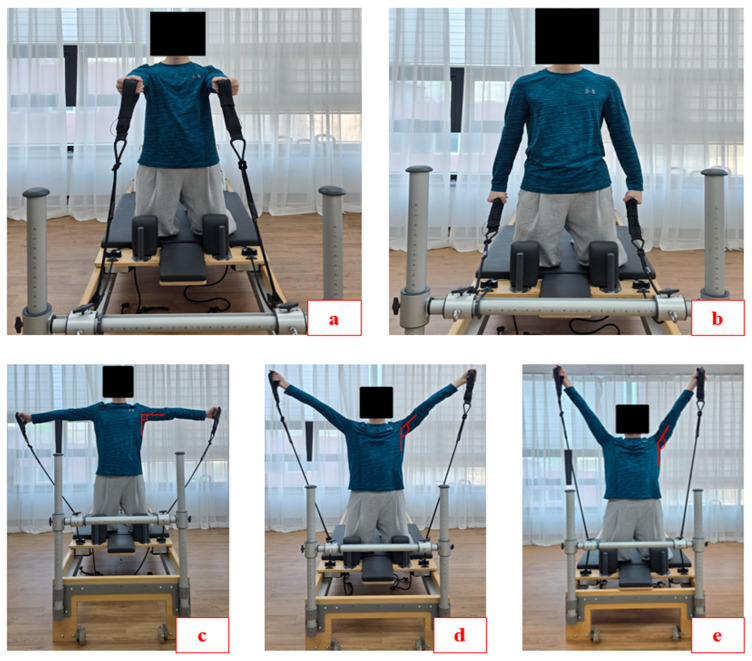
Pilates reformer arm work (AW) exercises. (**a**) Starting position, (**b**) performing position for AW0, (**c**) performing position for AW90, (**d**) performing position for AW135, (**e**) performing position for AW160.

Each participant repeated each Pilates reformer AW movement three times and was given a minimum rest time of 90 s. Additionally, at least 5 min rest periods were taken when the conditions (shoulder angle) changed. The abovementioned rest periods were the minimum rest times. Participants could rest longer between repetitions and sets as needed. To reduce order effects and fatigue bias, each condition was a randomized allocation with a permuted block design. The Pilates reformer AW sequence is divided into starting and performing positions. (1) Starting position (Figure 1a): One blue spring (normal length tension: 3–3.2 kg, stretched length tension: 0.67 kg/cm ± 5%) was hung on the reformer apparatus. The participants were set up in a half-kneeling position facing the pulley bar (or shoulder rest) on the carriage. Then, with both hands in a neutral position (not in pronation or supination), participants grasped the strap at the end of the spring-loaded rope and held it at 90 degrees of the shoulder flexion position. (2) Performing positions: ‘AW0’ (Figure 1b): Participants pulled the handle in the direction of the shoulder extension while maintaining the shape of the hand (neutral position) holding the strap (AW0-CON), held the shoulder at the 0-degree position (AW0-ISO), and returned to the starting position through shoulder flexion (AW0-ECC). ‘AW90’ (Figure 1c): Participants pulled the handle in the direction of shoulder horizontal abduction (like a T-shape with the torso and arms) while maintaining the shape of the hand holding the strap (AW90-CON), held the shoulder abduction at the 90-degree position (AW90-ISO), and returned to the starting position through shoulder horizontal adduction (AW90-ECC). ‘AW135’ (Figure 1d): Participants pulled the handle in the direction of shoulder flexion to achieve a 135-degree shoulder abduction position while maintaining the shape of the hand holding the strap (AW135-CON), held the shoulder abduction at the 135-degree position (AW135-ISO), and returned to the starting position through shoulder extension (AW135-ECC). ‘AW160’ (Figure 1e): Participants pulled the handle in the direction of shoulder flexion to achieve a 160-degree shoulder abduction position while maintaining the shape of the hand holding the strap (AW160-CON), held the shoulder abduction at the 160-degree position (AW160-ISO), and returned to the starting position through shoulder extension (AW160-ECC). In order to perform the various movements of the Pilates arm work (AW0, AW90, AW135, and AW160), we used a goniometer to establish the correct shoulder abduction angle for each participant. In addition, a mirror was placed in front of the reformer, and markers were placed at each angle to ensure that the correct angle was performed. If the participant did not perform at the designated angle, the test was discarded and performed again. To ensure consistent AW during the measurement, the test was performed to a metronome sound of 60 bpm with 2 s concentric, 2 s isometric, and 2 s eccentric time periods. A summary of the positions is presented in Table 1. To minimize any differences that may have occurred while performing the AW, participants were instructed to self-control their basic posture muscle contraction (CON-, ISO-, and ECC-phase) pace.

### 2.3. EMG Data Collection and Analysis

Surface EMG (sEMG) data were collected using a Biopac MP 160 device (Biopac System Inc., Goleta, CA, USA), and the sEMG data were analyzed at the IMSL. We processed the collected sEMG data for information on the muscle activation (concentric, isometric, and eccentric contraction phases) using AcqKnowledge 5.0 software (Biopac System Inc., Goleta, CA, USA). Surface electrodes were utilized using a multi-channel EMG system. The MP 160 device collected the following data information: sampling rate: 2000 Hz; common-mode rejection ratio (CMRR): >110 dB; input impedance > 10^12^ Ω; band-pass digital filter: 30 Hz (low cut-off) and 500 Hz (high cut-off). We used a disposable bipolar electrode (rectangle-shaped electrode, Ag/AgCl gel, 10 mm diameter). Before attaching the disposable electrode, the attachment site was shaved of hair, and the skin surface was abraded with a soft sanding sponge and cleaned with an alcohol swab. Two single disposable electrodes were attached to the muscle belly. The bipolar configuration and interelectrode distance should be at least 20 mm. Electrodes were attached to six muscles (upper trapezius, middle trapezius, lower trapezius, serratus anterior, posterior deltoid, and levator scapulae muscles) on the dominant side. The attachment location and maximum voluntary isometric contraction (MVIC) were based on the Surface Electromyography for Non-Invasive Assessment of Muscles (SENIAM) guidelines. Electrode sites were located as follows: The LS electrode was applied laterally to the C3–C4 spinous process, aligned between the posterior edge of the sternocleidomastoid and the anterior edge of the upper trapezius. The UT electrode was positioned at the midpoint between C7 and the acromion. The SA electrode was placed at the intersection of the mid-axillary line and the 7th intercostal space. The LT electrode was applied at the midpoint between the T7 spinous process and the inferior angle of the scapula. The MT electrode was placed 2 cm medial to the medial edge of the scapular spine. The PD electrode was affixed 2 cm below the posterior aspect of the acromion. The ground electrodes were placed on the bilateral acromion, olecranon, and inferior angles of the scapula. The MVIC was measured as follows: LS, UT, and SA activities were measured in the sitting position, while MT, LT, and PD activities were assessed in the prone position. For LS assessment, the neck was rotated toward the ipsilateral side with the shoulder elevated [22]. UT assessment required contralateral neck rotation, ipsilateral flexion, and shoulder elevation [23]. SA activity was measured with the shoulder positioned at 135° flexion [24]. MT and PD muscle activations were evaluated on shoulder-abducted arm to 90° with shoulder horizontal abduction in prone position. LT assessment was conducted with the shoulder abducted to 135° and the thumb oriented upward [25]. The raw sEMG signal data were smoothed with the root mean square (RMS) method using a sliding window (100 m/s). The data collected were analyzed to determine the level of the normalized amplitude of each muscle activity (%MVIC) during the 3 phases of AW movement. The results used the mean value of the muscle activation data for each phase during the AW movement performance. The 3 trial data points collected from each participant were averaged. All data samples had the same processing.

### 2.4. Statistical Analyses

Statistical analyses were conducted using IBM SPSS version 28.0 (IBM SPSS, Armonk, NY, USA). All sEMG numerical variables were tested for normal distribution using the Shapiro–Wilk test. For each contraction phase (concentric, isometric, eccentric), a repeated-measures analysis of variance (RM-ANOVA) was used to compare the differences in the normalized muscle activation (mean %MVIC) and muscle activity ratio between the AW movement conditions. Post hoc analysis was performed using the Bonferroni correction method. When Mauchly’s test indicated a violation of sphericity, correction factors were applied. The Greenhouse–Geisser correction was used when the average of the two epsilon values was <0.75; the Huynh–Feldt correction was applied when the average was ≥0.75. A significance level of *p* < 0.05 was set for all statistical tests.

## 3. Results

The results of the analysis of scapular muscle activation (concentric, isometric, and eccentric phases) according to the difference in the shoulder abduction angle during the Pilates reformer AW movement are presented in Table 2. All muscles (LS, MD, PD, UT, MT, LT, and SA) had statistically significant differences in each phase of contraction (*p* < 0.001).

**Figure 2:**
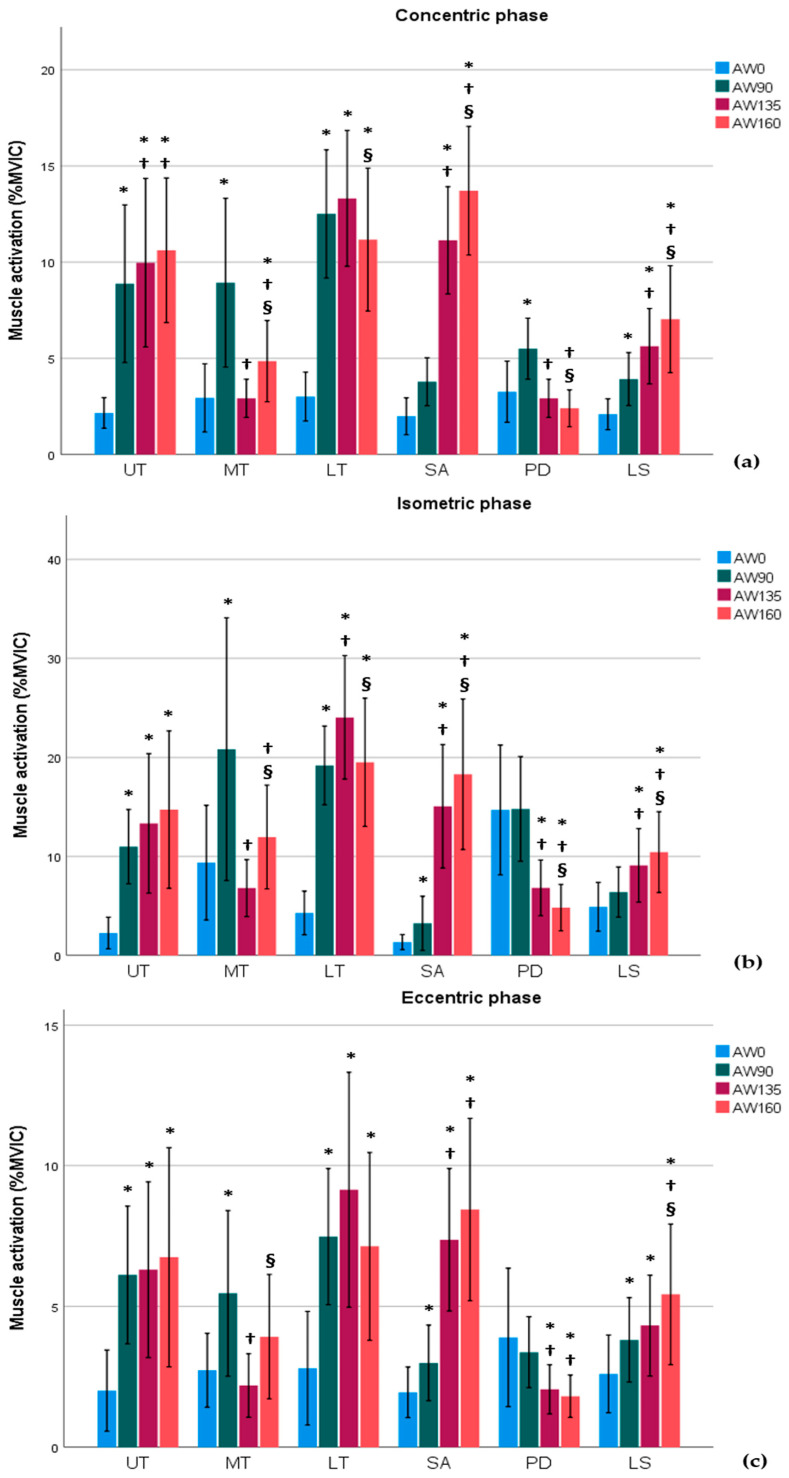
The mean (standard deviation) scapular muscle activity (%MVIC) at each arm position angle (0°, 90°, 135°, and 160°) and in concentric (**a**), isometric (**b**), and eccentric (**c**) phases during AW. Color shading of the bars corresponds to the shoulder abduction angle (0°: blue; 90°: green; 135°: burgundy; 160°: orange). On the *X*-axis, the plane of scapular muscles is represented as follows: UT, upper trapezius; MT, middle trapezius; LT, lower trapezius; SA, serratus anterior; PD, posterior deltoid; LS, levator scapulae. The lettering scheme is used to denote significantly different means (*p* < 0.05). * Conditions that showed a significant difference from 0° AW angle; ^†^ conditions that showed a significant difference from 90° AW angle; ^§^ conditions that showed a significant difference from 135° AW angle.

The muscle activation during the concentric phase is shown in Figure 2a. In the UT, the activation was the highest at 135° and 160°, followed by at 90° and 0°. The MT showed the highest activation at 90°, followed by at 160°, with the lowest activation at 0° and 135°. The LT had higher activation at 90° than at 0°, and higher activation at 160° than at 0°, but lower activation at 90° compared to at 160°. The SA exhibited increasing activation as the shoulder angle increased. The PD had higher activation at 90° than at 0°, followed by at 135° and 160°. The LS showed higher activation as the shoulder angle increased.

In the isometric phase, the muscle activation was as follows (Figure 2b). In the UT, the activation was lower at 0° compared to at the other angles. The MT showed higher activation at 90° than at 0°, higher activation at 160° than at 135°, and higher activation at 135° than at 160°. The LT had the highest muscle activation at 135°, followed by at 90° and 160°, and the lowest at 0°. The SA had increased muscle activation as the shoulder angle increased. The PD had the highest muscle activation at 0° and 90°, followed by at 135° and 160°, and the lowest at 0°. The LS had the highest muscle activation at 160°, followed by at 135°, and the lowest at 0° and 90°.

The muscle activation during the eccentric phase was as follows (Figure 2c). In the UT, the activation was lower at 0° compared to at the other angles. The MT showed higher activation at 90° than at 0°, higher activation at 160° than at 135°, and higher activation at 135° than at 160°. The LT had the highest activation at 135°, followed by at 90° and 160°, with the lowest activation at 0°. The SA exhibited increased activation as the shoulder angle increased. The PD showed the highest activation at 0° and 90°, followed by at 135° and 160°, with the lowest activation at 160°. The LS had the highest activation at 160°, followed by at 135°, and the lowest at both 0° and 90°.

Table 2 and Figure 2 present the results of analyzing the differences in the muscle activity ratios during the Pilates reformer AW exercise. Statistically significant differences were observed in the UT/LT, UT/SA, LS/LT, and LS/SA muscle ratios (*p* < 0.001). The UT/LT activity ratio was lower at 90° and 135° compared to at 160°. The UT/SA ratio was lower at 0°, 135°, and 160° compared to at 90°. The LS/LT ratio was the highest at 0°, followed by at 160°, and the lowest at 90° and 135°. The LS/SA ratio was lower at 135° and 160° compared to at 0° and 90°.

**Figure 3:**
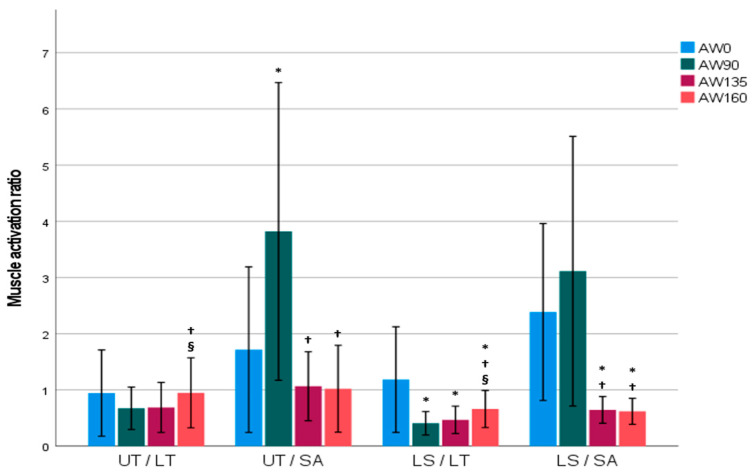
The mean (standard deviation) scapular muscle activity ratio at each arm position angle (0°, 90°, 135°, 160°) during AW. Color shading of the bars corresponds to the shoulder abduction angle (0°: blue; 90°: green; 135°: burgundy; 160°: orange). On the *X*-axis, the plane of scapular muscles is represented as follows: UT, upper trapezius; LT, lower trapezius; SA, serratus anterior; LS, levator scapulae. The lettering scheme is used to denote significantly different means (*p* < 0.05). * Conditions that showed a significant difference from 0° AW angle; ^†^ conditions that showed a significant difference from 90° AW angle; ^§^ conditions that showed a significant difference from 135° AW angle.

Table 3 and Figure 3 present the results of analyzing the differences in the muscle activity ratios during the Pilates reformer AW exercise. Statistically significant differences were observed in the UT/LT, UT/SA, LS/LT, and LS/SA muscle ratios (*p* < 0.001). The UT/LT activity ratio was lower at 90° and 135° compared to at 160°. The UT/SA ratio was lower at 0°, 135°, and 160° compared to at 90°. The LS/LT ratio was the highest at 0°, followed by at 160°, and the lowest at 90° and 135°. The LS/SA ratio was lower at 135° and 160° compared to at 0° and 90°.

## 4. Discussion

This study aimed to investigate muscle activation in the UT, MT, LT, SA, PD, and LS muscles, as well as the LS/LT, UT/LT, LS/SA, and UT/SA muscle activity ratios, during the Pilates reformer AW movement at different shoulder angles (0°, 90°, 135°, and 160°). Electromyographic data revealed that the UT, LT, SA, PD, and LS showed the lowest activation at 0°. The UT, SA, and LS demonstrated increased activation as the shoulder angle increased. Interestingly, the LT had the highest activation at 135°. The MT activation was the highest at 90°. The PD exhibited higher activation at 0° and 90° compared to at the other angles.

The UT muscle activation was the highest at the shoulder abduction angle of 160° across all phases, compared to at 0°, 90°, and 135°. Previous studies have shown that UT activation is higher at shoulder angles of 90° and 150° compared to at 30° during shrug exercises [13]. Similar to our findings, when performing scapular retraction with an elastic resistance band, the UT activity increased at the shoulder angles of 45°, 90°, and 120° compared to at 0° [16]. Other studies have also reported that upper trapezius activity consistently increases as the shoulder abduction angle surpasses 120° [8]. These results suggest that the shoulder abduction angle, rather than the exercise intensity or method, significantly impacts UT activation during upper extremity exercises. Persistent overactivity of the UT can lead to scapular dysfunction (or dyskinesia), such as subacromial impingement or glenohumeral instability [26,27]. Therefore, when performing AW at higher shoulder abduction angles, care should be taken to avoid the excessive activation of the UT. A lower UT/LT activation ratio is generally preferred in rehabilitation exercises because it indicates reduced UT dominance and better activation of the LT, which contributes to proper scapular positioning and stability [16]. Similarly, a lower UT/SA ratio suggests decreased UT overuse and increased engagement of the SA, which plays a crucial role in scapular upward rotation and stabilization [28]. Our results indicate that performing AW at a 135° shoulder abduction angle may be optimal, as this position minimizes UT overactivation while promoting the higher activation of both the LT and SA, facilitating improved scapular control and shoulder function.

The MT muscle activation was the highest at a shoulder abduction angle of 90° across all phases, compared to at 0°, 135°, and 160°. Previous research has shown that during prone horizontal abduction, middle trapezius activation is higher at 90° than at 125° and 160° [29]. Another study found that during shoulder abductions with light dumbbells, the muscle activation increased from 0° to 90° but decreased from 90° to 160° [30]. In our study, the light spring resistance during the AW likely explains the similar results. The higher activation of the MT at 90° compared to at other angles is due to the reduction in the MT’s moment arm at shoulder abduction angles above 100° [31]. Additionally, optimal MT recruitment occurs when UT activity is minimized [32]. Therefore, performing AW at 90° is considered optimal for maximizing MT activation.

The LT exhibited higher muscle activation at a shoulder abduction angle of 135° across all contraction phases, compared to at 0°, 90°, and 160°. The LT functions as a scapular stabilizer by facilitating scapular upward rotation and depression during shoulder abduction [33]. It also shows increased activation when the shoulder abduction angle exceeds 90° [34]. In a previous study, the LT activation was the highest at 120° of shoulder abduction compared to at 30°, 60°, and 90° [15]. While prior research used the prone position to induce LT activity, our study, which utilized the kneeling position, found similar results, suggesting that the shoulder abduction angle is the primary factor influencing LT muscle activation. Researchers recommend shoulder abduction near 145°, aligning with the muscle fiber direction, for maximum LT activation. However, there is no clear angle-specific analysis [35,36,37]. In our study, we analyzed shoulder abduction angles of 135° and 160° to determine the posture that maximizes LT muscle activity. The results indicated that the LT activation was the highest at a 135° shoulder abduction angle, with excessively high angles leading to a decrease in muscle activation.

The SA muscle activation was the highest at 160° across all contraction phases, compared to at 0°, 90°, and 135°. The SA plays a stabilizing role by preventing scapular anterior tilt through upward rotation and protraction during arm elevation [35,38,39]. As such, SA activation is typically observed at shoulder abduction angles of 90° or greater, with some degree of scapular upward rotation [40]. For the manual muscle testing of the SA, shoulder abduction between 120° and 130° is recommended [41]. In a previous study, the SA activity was approximately 155% higher at shoulder abduction angles above 120° compared to at angles below 80° [34]. Other studies have reported a progressive increase in SA activation between 90° and 140° of shoulder elevation [14]. While earlier studies primarily examined SA activity at angles below 140°, our study found that the SA activation was significantly higher at a shoulder abduction of 160°. These results suggest that increasing the shoulder abduction angle could be an effective strategy to enhance SA muscle activation.

The PD muscle activation was the highest at 90° compared to at 0°, 135°, and 160° across all contraction phases. The PD is clinically important in normal shoulder function, as it contributes to shoulder external rotation alongside the rotator cuff muscles [42]. Previous studies have shown that PD activation is the highest during rowing movements at a shoulder angle of 90°, with the muscle activity gradually decreasing as the shoulder angle increases [18]. Similarly, our study found that the PD muscle activation decreased as the shoulder angle increased from 90° to 135° and 160°. Additionally, PD is associated with a reduction in lever arm strength when the shoulder elevation exceeds 120°, due to a decrease in the effective length of the lever arm [43]. Moreover, PD activation is reported to be maximal when the shoulder is in full external rotation [44]. Based on these findings, performing the movement at a shoulder angle of 90° with full external rotation is recommended for optimal PD training.

The LS muscle activation was the highest at 160° compared to at 0°, 90°, and 135° across all contraction phases. Consistent with our findings, previous studies have reported increased LS activity at 90° and 150° of humeral elevation compared to at 30° in the scapular plane [14]. Additionally, LS muscle activation generally increases as the shoulder abduction angle rises [13]. This increase is attributed to scapular upward rotation, which enhances LS activation [45,46]. However, the LS is responsible for scapular superior angle elevation and medial rotation [47], and excessive activation of the LS can disrupt proper scapular movement [48]. A lower LS/LT activation ratio is generally preferred, as it indicates the reduced dominance of the LS, allowing the LT to effectively facilitate scapular depression and upward rotation, which are essential for maintaining proper shoulder mechanics [49]. Similarly, a lower LS/SA ratio suggests the increased activation of the SA, which plays a crucial role in stabilizing the scapula against the thoracic wall and promoting normal scapular kinematics [50]. Our findings suggest that performing AW at a shoulder abduction angle of 135° may be optimal, as this position minimizes LS overactivation while enhancing LT and SA engagement, thereby supporting proper scapular movement and stabilization.

Previous studies have shown that the magnitudes of the muscle activation in the concentric and eccentric phases are similar [51]. However, studies analyzing the isometric phase have reported significantly higher levels of muscle activation [52]. During the isometric phase, motor units are mobilized to maintain a constant force, resulting in higher muscle activity [53]. We believe that the higher activation observed in the isometric phase, compared to in the concentric and eccentric phases, is due to our analysis using the mean %MVIC value of muscle activation over the duration of the movement. Therefore, focusing on the isometric phase is essential for maximizing the activation of the targeted muscles when performing AW movements.

Our study has several limitations. First, our study only included men in their 20s, which may limit the direct generalizability of the findings to women or mixed groups. It is possible that the physiological and biomechanical effects of Pilates may differ between genders. Therefore, future studies should recruit women, other age groups, or subjects with musculoskeletal disorders of the shoulder region to analyze muscle activity to generalize the results. Second, because the muscle activity was analyzed by performing movements on only one spring, there is a limitation in identifying the effect of the movement on the intensity. Future studies should investigate muscle activity at different resistance levels of resistance springs to further refine exercise recommendations. Third, this study was limited to the muscles associated with the scapular region, so future studies should further analyze the activation of muscles associated with the upper extremities and shoulders. Finally, since this study only measured scapular muscle activation, it is difficult to determine the actual movement of the scapula. Therefore, future studies should directly measure and analyze the position of the scapula.

## 5. Conclusions

Our results highlight the importance of selecting an appropriate shoulder abduction angle during the AW movement to optimize scapular muscle activation. An abduction angle of 135° during the AW movement effectively promotes LT and SA activation, while the UT and LS show relatively low activation levels, which is beneficial for improving scapular stability. Clinicians and instructors can use these results to prescribe specific abduction angles to target key scapular muscles, such as utilizing 135° abduction to decrease the activation of the UT and LS and increase the activation of the LT and SA to prevent shoulder instability or scapular dyskinesia.

## Figures and Tables

**Table 1:** Arm work position.

Position	Description
Starting position	Half-kneeling, facing pulley bar, hands in neutral, shoulder flexion held at 90°
Arm work, 0° (AW0)	Starting position, pull handle in shoulder extension direction 0° (2 s), hold (2 s), return to starting position (2 s)
Arm work, 90° (AW90)	Starting position, pull handle in horizontal abduction 90° (2 s), hold (2 s), return to starting position (2 s)
Arm work, 135° (AW135)	Starting position, pull handle in shoulder abduction 135° (2s), hold (2 s), return to starting position (2 s)
Arm work, 160° (AW160)	Starting position, pull handle in shoulder abduction 160° (2 s), hold (2 s), return to starting position (2 s)

**Table 2:** Muscle activity in each contraction phase according to each angle during AW.

Variable		Pilates Reformer AW Shoulder Angle [95% CI]				*F* (*p*)	*η*_p_^2^	Post Hoc
		0°	90°	135°	160°			
UT	CON	2.25 ± 1.43 [1.63; 2.87]	7.40 ± 3.95 * [5.69; 9.11]	9.99 ± 5.20 *^,†^ [7.74; 12.24]	10.94 ± 5.55 *^,†^ [8.53; 13.34]	54.704 (<0.001)	0.713	0 < 90 < 135, 160
	ISO	2.26 ± 1.60 [1.55; 2.97]	10.99 ± 3.75 * [9.33; 12.66]	13.34 ± 7.04 * [10.22; 16.47]	14.73 ± 7.95 * [11.21; 18.26]	43.659 (<0.001)	0.675	0 < 90, 135, 160
	ECC	2.01 ± 1.44 [1.37; 2.65]	6.12 ± 2.44 * [5.04; 7.20]	6.30 ± 3.11 * [4.92; 7.69]	6.75 ± 3.89 * [5.02; 8.47]	35.217 (<0.001)	0.626	0 < 90, 135, 160
MT	CON	3.47 ± 1.87 [3.38; 4.29]	10.49 ± 4.22 * [8.61; 12.36]	3.27 ± 1.26 ^†^ [2.71; 3.83]	5.90 ± 3.02 *^,†,§^ [4.56; 7.24]	39.321 (<0.001)	0.652	0, 135 < 160 < 90
	ISO	9.38 ± 5.80 [6.87; 11.89]	20.83 ± 13.26 * [15.10; 26.56]	6.81 ± 2.87 ^†^ [5.57; 8.05]	11.96 ± 5.24 ^†,§^ [9.70; 14.23]	15.552 (<0.001)	0.414	0 < 90 135 < 160 < 90
	ECC	2.74 ± 1.31 [2.15; 3.32]	5.16 ± 2.94 * [4.16; 6.76]	2.19 ± 1.13 ^†^ [1.69; 2.69]	3.93 ± 2.21 ^§^ [2.95; 4.90]	13.698 (<0.001)	0.395	0, 135 < 90 135 < 160
LT	CON	3.22 ± 1.38 [2.63; 3.82]	11.92 ± 3.29 * [10.50; 13.34]	13.78 ± 3.66 * [12.20; 15.37]	10.89 ± 2.77 *^,§^ [9.69; 12.09]	106.430 (<0.001)	0.829	0 < 90 0 < 160 < 135
	ISO	4.30 ± 2.21 [3.36; 5.23]	19.20 ± 3.98 * [17.52; 20.87]	24.05 ± 6.22 *^,†^ [21.42; 26.68]	19.52 ± 6.48 *^,§^ [16.78; 22.25]	95.503 (<0.001)	0.806	0 < 90, 160 < 135
	ECC	2.80 ± 2.01 [1.91; 3.70]	7.48 ± 2.42 * [6.41; 8.55]	9.14 ± 4.18 * [7.29; 11.00]	7.13 ± 3.33 * [5.65; 8.61]	23.957 (<0.001)	0.533	0 < 90, 160, 135
SA	CON	1.98 ± 0.77 [1.64; 2.32]	3.96 ± 1.63 * [3.24; 4.68]	10.32 ± 3.33 *^,†^ [8.84; 11.80]	13.00 ± 4.45 *^,†,§^ [11.47; 14.53]	149.709 (<0.001^)^	0.877	0 < 90 < 135 < 160
	ISO	1.35 ± 0.76 [1.03; 1.67]	3.24 ± 2.74 * [2.09; 4.40]	15.07 ± 6.24 *^,†^ [12.44; 17.70]	18.31 ± 7.61 *^,†,§^ [15.09; 21.52]	83.831 (<0.001)	0.785	0 < 90 < 135 < 160
	ECC	1.95 ± 0.90 [1.56; 2.34]	2.99 ± 1.34 * [2.42; 3.57]	7.37 ± 2.53 *^,†^ [6.27; 8.46]	8.44 ± 3.24 *^,†^ [7.04; 9.84]	62.538 (<0.001)	0.740	0 < 90 < 135, 160
PD	CON	3.53 ± 1.66 [2.81; 4.25]	5.35 ± 1.82 * [4.56; 6.14]	3.20 ± 1.20 ^†^ [2.68; 3.71]	2.60 ± 1.19 ^†,§^ [2.09; 3.12]	25.423 (<0.001)	0.536	0 < 90 160 < 135 < 90
	ISO	14.70 ± 6.56 [11.93; 17.48]	14.80 ± 5.29 [12.57; 17.03]	6.83 ± 2.81 *^,†^ [5.64; 8.01]	4.83 ± 2.35 *^,†,§^ [3.84; 5.82]	48.885 (<0.001)	0.680	160 < 135 < 0, 90
	ECC	3.38 ± 1.26 [2.83; 3.92]	3.90 ± 2.45 [2.84; 4.96]	2.06 ± 0.87 *^,†^ [1.68; 2.44]	1.81 ± 0.75 *^,†^ [1.49; 2.14]	13.276 (<0.001)	0.376	135, 160 < 0, 90
LS	CON	2.12 ± 0.89 [1.76; 2.50]	3.93 ± 1.37 * [3.36; 4.51]	5.74 ± 1.90 *^,†^ [4.94; 6.54]	6.90 ± 2.36 *^,†,§^ [5.90; 7.89]	80.954 (<0.001)	0.779	0 < 90 < 135 < 160
	ISO	4.91 ± 2.46 [3.85; 5.98]	6.40 ± 2.54 [5.31; 7.50]	9.10 ± 3.71 *^,†^ [7.50; 10.71]	10.44 ± 4.08 *^,†,§^ [8.67; 12.21]	27.688 (<0.001)	0.557	0, 90 < 135 < 160
	ECC	2.61 ± 1.38 [2.00; 3.22]	3.81 ± 1.49 * [3.15; 4.48]	4.32 ± 1.79 * [3.53; 5.11]	5.43 ± 2.49 *^,†,§^ [4.21; 6.53]	25.982 (<0.001)	0.553	0 < 90, 135 < 160

Values (mean %MVIC) are presented as mean ± standard deviation. AW, arm work; CON, concentric contraction phase; ISO, isometric contraction phase; ECC, eccentric contraction phase; UT, upper trapezius; MT, middle trapezius; LT, lower trapezius; SA, serratus anterior; PD, posterior deltoid; LS, levator scapulae; * conditions that showed a significant difference from 0°; ^†^ conditions that showed a significant difference from 90°; ^§^ conditions that showed a significant difference from 135°.

**Table 3:** Muscle activity ratio according to each angle during AW.

Variable	Pilates Reformer AW Shoulder Angle [95% CI]				*F* (*p*)	*η*_p_^2^	Post Hoc
	0°	90°	135°	160°			
UT/LT	0.94 ± 0.77 [0.60; 1.29]	0.67 ± 0.38 [0.51; 0.84]	0.69 ± 0.45 [0.49; 0.89]	0.95 ± 0.62 ^†,§^ [0.67; 1.23]	4.152 (0.032)	0.165	90, 135 < 160
UT/SA	1.72 ± 1.47 [1.06; 2.37]	3.82 ± 2.65 ^*^ [2.65; 4.99]	1.07 ± 0.61 ^†^ [0.79; 1.34]	1.02 ± 0.77 ^†^ [0.79; 1.34]	19.104 (<0.001)	0.476	0, 135, 160 < 90
LS/LT	1.18 ± 0.94 [0.79; 1.58]	0.41 ± 0.21 * [0.32; 0.50]	0.47 ± 0.24 * [0.36; 0.57]	0.66 ± 0.33 *^,†,§^ [0.52; 0.80]	15.192 (<0.001)	0.398	90, 135 < 160 < 0
LS/SA	2.39 ± 1.57 [1.70; 3.07]	3.11 ± 2.40 [2.08; 4.15]	0.65 ± 0.24 *^,†^ [0.54; 0.75]	0.62 ± 0.23 *^,†^ [0.52; 0.72]	21.347 (<0.001)	0.492	135, 160 < 0, 90

Values (mean %MVIC) are presented as mean ± standard deviation. AW, arm work; CON, UT, upper trapezius; LT, lower trapezius; LS, levator scapulae; SA, serratus anterior; * conditions that showed a significant difference from 0°; ^†^ conditions that showed a significant difference from 90°; ^§^ conditions that showed a significant difference from 135°.

## Data Availability

The data for this study are available from the corresponding authors upon reasonable request.
